# MC180295 is a highly potent and selective CDK9 inhibitor with preclinical in vitro and in vivo efficacy in cancer

**DOI:** 10.1186/s13148-023-01617-3

**Published:** 2024-01-03

**Authors:** Hanghang Zhang, Chen Huang, John Gordon, Sijia Yu, George Morton, Wayne Childers, Magid Abou-Gharbia, Yi Zhang, Jaroslav Jelinek, Jean-Pierre J. Issa

**Affiliations:** 1 Fels Institute for Cancer Research, Lewis Katz School of Medicine at Temple University, Philadelphia, PA 19140 USA; 2 Moulder Center for Drug Discovery Research, Temple University School of Pharmacy, Philadelphia, PA 19140 USA; 3 Coriell Institute for Medical Research, 403 Haddon Avenue, Camden, NJ 08103 USA; 4 Center for Discovery and Innovation, Hackensack Meridian Health, Nutley, NJ 07110 USA; 5 Cooper Medical School at Rowan University, Camden, NJ 08103 USA; 6 Department of Internal Medicine, University of Texas Southwestern Medical Center, Dallas, TX 75390 USA

**Keywords:** Epigenetic therapy, CDK9, Immunosensitization, MC180380, Anti-tumoral effects

## Abstract

**Background:**

Inhibition of cyclin-dependent kinase 9 (CDK9), a novel epigenetic target in cancer, can reactivate epigenetically silenced genes in cancer by dephosphorylating the SWI/SNF chromatin remodeler BRG1. Here, we characterized the anti-tumor efficacy of MC180295, a newly developed CDK9 inhibitor.

**Methods:**

In this study, we explored the pharmacokinetics of MC180295 in mice and rats, and tested the anti-tumor efficacy of MC180295, and its enantiomers, in multiple cancer cell lines and mouse models. We also combined CDK9 inhibition with a DNA methyltransferase (DNMT) inhibitor, decitabine, in multiple mouse models, and tested MC180295 dependence on T cells. Drug toxicity was measured by checking body weights and complete blood counts.

**Results:**

MC180295 had high specificity for CDK9 and high potency against multiple neoplastic cell lines (median IC50 of 171 nM in 46 cell lines representing 6 different malignancies), with the highest potency seen in AML cell lines derived from patients with MLL translocations. MC180295 is a racemic mixture of two enantiomers, MC180379 and MC180380, with MC180380 showing higher potency in a live-cell epigenetic assay. Both MC180295 and MC180380 showed efficacy in in vivo AML and colon cancer xenograft models, and significant synergy with decitabine in both cancer models. Lastly, we found that CDK9 inhibition-mediated anti-tumoral effects were partially dependent on CD8 + T cells in vivo, indicating a significant immune component to the response.

**Conclusions:**

MC180380, an inhibitor of cyclin-dependent kinase 9 (CDK9), is an efficacious anti-cancer agent worth advancing further toward clinical use.

**Supplementary Information:**

The online version contains supplementary material available at 10.1186/s13148-023-01617-3.

## Background

Epigenetic regulators of gene silencing are validated targets in cancer [1]. One goal of epigenetic therapy is to reactivate silenced tumor suppressor genes, to facilitate cancer cell redifferentiation and apoptosis. Another therapeutic benefit of epigenetic therapy is induction of an immuno-sensitizing interferon response, mediated in part by reactivation of repetitive elements [2]. Based on this concept, several DNA methyltransferase (DNMT) and histone deacetylase (HDAC) inhibitors have been approved by the FDA to treat hematological malignancies [1]. However, epigenetic treatment options remain limited, with no such drugs approved for solid tumors.

Cyclin-dependent kinases (CDKs) belong to a family of serine/threonine kinases that associate with the cell cycle (CDKs 1, 2, 4 and 6) and gene transcription (CDKs 7, 8, 9, 12, and 13) [3]. CDKs have been studied extensively as potential targets in cancer, resulting in the development of multiple CDK inhibitors. Cyclin-dependent kinase 9 (CDK9), the catalytic subunit of P-TEFb, is a positive transcriptional elongation regulator recruited to phosphorylate the C-terminal domain of RNA polymerase II (RNAPII) on serine-2. P-TEFb mediates phosphorylation of the C-terminal repeat domain of the (DRB)- sensitivity-induced factor (DSIF) and negative elongation factor (NELF) that associate with RNAPII, thus releasing the RNA polymerase from inhibition by these proteins [4, 5]. CDK9 is important for RNAPII-mediated transcription elongation, and the expression of specific gene sets has been shown to be highly sensitive to CDK9 inhibition in cancer cells, including genes encoding mRNA transcripts with high turnover rates for anti-apoptotic and pro-survival proteins (e.g., MYC, and MCL1), highly expressed genes, fusion chimeras (e.g., MYC, MLL1-AF9), and genes associated with super-enhancers [6].

Previously, we unexpectedly found that CDK9 inhibition also reactivates epigenetically silenced genes in cancer. Using an unbiased phenotype-based screen, we identified CDK9 as a novel epigenetic repressor that mediates gene silencing via phosphorylating the SWI/SNF chromatin-remodeling protein, BRG1. Moreover, we showed that CDK9 inhibition activates an interferon (IFN) response, endogenous retroviruses, and immunosensitizes cancer cells to the checkpoint inhibitor anti-PD1, in vivo [7]. Thus, CDK9 inhibition not only represses oncogenes, but also reactivates silenced tumor suppressor genes and induces tumor cell immune responses, making it a promising epigenetic therapy. Previously, we developed a novel and potent CDK9 inhibitor – MC180295 [7]. In this study, we extensively characterize MC180295 and its enantiomers, explore its pharmacokinetics, and demonstrate its broad anti-tumor efficacy, in vitro and in vivo.

## Results

### MC180295 is a potent and selective CDK9 inhibitor with antitumor effects against multiple cancer cell lines

**Fig. 1:**
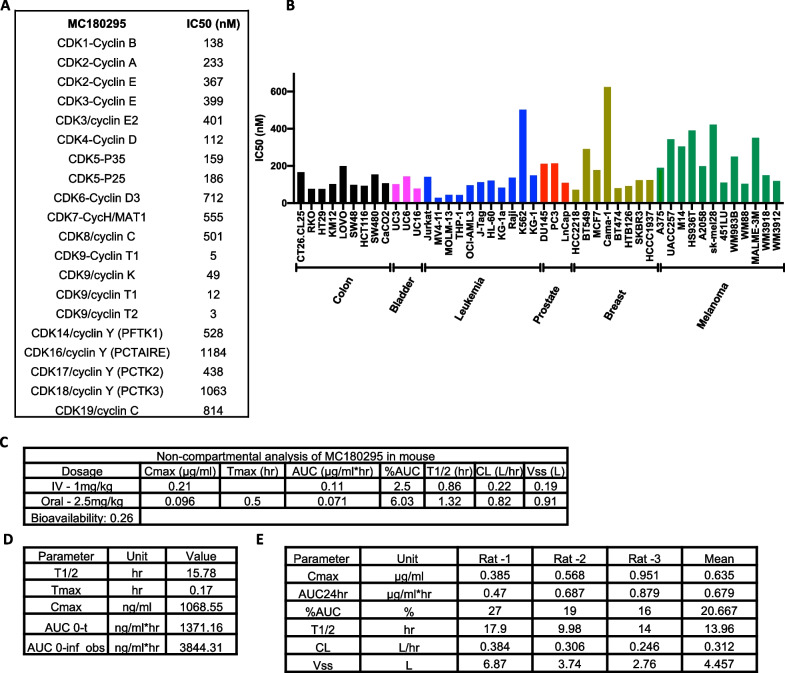
MC180295 enzymatic/growth inhibition and pharmacokinetics. **A** In vitro activity (IC50 values) of MC180295 against a panel of CDKs. **B** MC180295 IC50 values against 46 cell lines from 6 different cancer types. **C** Pharmacokinetic analyses after oral or IV administration of low-dose (1 mg/kg IV or 2.5 mg/kg oral) MC180295 to mice. **D** Pharmacokinetic analyses after IP administration of 10 mg/kg MC180295 to mice. **E** Pharmacokinetic analyses after IV administration of 1 mg/kg MC180295 to rats

To identify novel epigenetic targets, we previously used a phenotype-based screen, the YB5 SW48 daughter cell line reporter system, to identify drugs that can reactivate GFP silenced by a methylated CMV promoter [8]. Using this screen, we developed and optimized a potent and selective CDK9 inhibitor, MC180295, and showed its significant anti-proliferative effects in cancer cell lines, with minimal effects on normal cells [7]. To further characterize MC180295, we first tested its inhibitory activity against a comprehensive panel of CDKs (Fig. 1A), finding that MC180295 is highly potent and selective toward CDK9/cyclin T (IC50 3–12 nM, based on two independent assays). To better understand MC180295's antitumor effects, we tested its growth inhibition in a panel of cancer cell lines. We found that it inhibited cell growth in multiple cancer cell lines (median IC50 171 nM), with highest potency against AML cell lines derived from patients with MLL translocations (MV4-11, MOLM-13 and THP-1) (Fig. 1B and Additional file 1: Fig. S1A).

We next studied the pharmacokinetics (PK) of MC180295. At an IV dose of 1 mg/kg (Fig. 1C and Additional file 1: Fig. S1B), it had a relatively short half-life (0.86 h), while an oral dose of 2.5 mg/kg MC180295 (Fig. 1C and Additional file 1: Fig. S1B) yielded a half-life of 1.3 h and bioavailability of 26%. Given IP at 10 mg/kg (Fig. 1D and Additional file 1: Fig. S1C), MC180295's half-life was much longer (15.8 h), suggesting a potential compartmental effect. Interestingly, its half-life was also long (~ 14 h) when given IV to rats (Fig. 1E and Additional file 1: Fig. S1D).

### Characterization of the MC180295 enantiomers

**Fig. 2:**
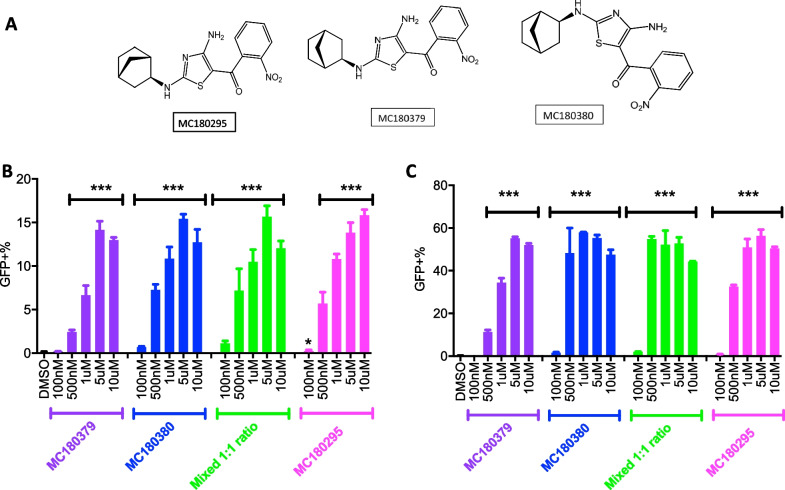
MC180295 and its enantiomers, MC180379 and MC180380, ability to epigenetically reactivate GFP expression in the YB5 reporter system. **A** Structures of MC180295 and its enantiomers, MC180379 and MC180380. Expression of GFP (measured by FACS), **B** 24 h, and **C** 4 days, after a one-time treatment of YB5 cells with MC180379, MC180380, MC180295, and a one-to-one mixture of MC180379 and MC180380. Data are shown as means ± SDs, n = 3. *p < 0.05, ***p < 0.001

MC180295 is composed of two enantiomers. We separated and purified the two enantiomers – MC180379 and MC180380 (Fig. 2A) for testing in YB5 cells, finding MC180380 to be more potent than MC18379 in inducing GFP. MC180380 first reactivated GFP at 100 nM, after 24 h treatment, compared to 500 nM MC180379 (Fig. 2B). Consistent with our previous result [7], MC180295 activated GFP at 100 nM, after 24-h treatment. We then mixed MC180379 and MC180380 at a one-to-one ratio and found that the lowest concentration reactivating GFP after mixing was 100 nM (Fig. 2B).

Previously, we showed that the expression levels of epigenetically silenced CDK9 targets peaked 4 days after dosing MC180295 once on day one. Consistent with our 24-h result, 4 days after a one-time drug exposure showed better GFP reactivation, with lower effective MC180380 doses (Fig. 2C), and an independent imaging reading experiment, with less sensitivity than FACS, replicated these findings (Additional file 1: Fig. S2A). Thus, MC180380 is epigenetically more potent than MC180379. Importantly, we previously showed that within nanomolar ranges, MC180295 was highly selective against CDK9, without affecting downstream targets of other CDKs, in YB5 cells [7].

### MC180380 shows promising antitumor efficacy in vivo

**Fig. 3:**
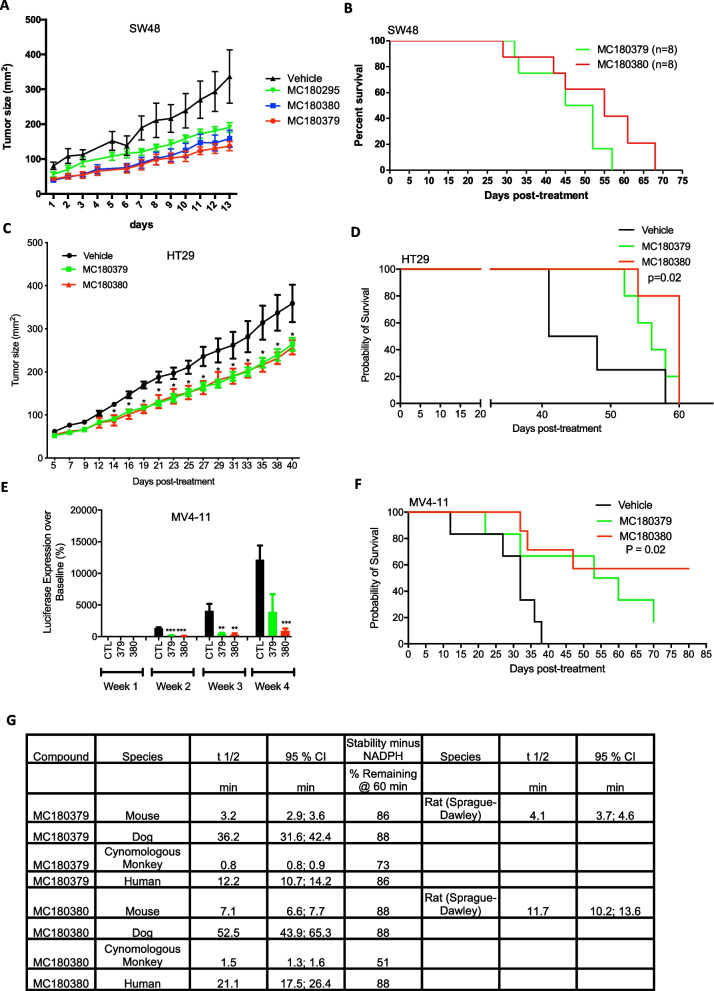
Antitumor, survival, and metabolic effects of MC180295 and its enantiomers, MC180379 and MC180380. **A** Antitumor effects of MC180295, MC180379, and MC180380. NSG mice were inoculated (s.c.) with 2 × 10^6^ SW48 cells. Eleven days later, when tumors were palpable, 20 mg/kg MC180295, MC180379, MC180380, or vehicle were administered (i.p.) every other day (q.o.d.). Tumor sizes were measured using a caliper. **B** SW48 mouse survival with q.o.d. i.p administration of 20 mg/kg MC180379 or MC180380. **C** Antitumor effects of MC180379 and MC180380. NSG mice were inoculated (s.c.) with 1 × 10^6^ HT29 cells. Twelve days later, when tumors were palpable, 20 mg/kg MC180379, MC180380, or vehicle were administered (i.p.) q.o.d. Tumor sizes were measured using a caliper. **D** HT29-xenografted mouse survival following (i.p.) q.o.d. dosing with 20 mg/kg MC180379 or MC180380. **E** NSG mice were inoculated (i.p.) with 5 × 10^5^ MV4-11-luc cells. Four days later, when substantial tumor burden was evident by bioluminescence imaging, MC180379, MC180380, or vehicle were administered (i.p.) q.o.d at 20 mg/kg. Luciferase expression was quantified and calculated. **F** Mouse survival in days. MC180380 significantly extended survival in i.p. MV4-11 model mice. **G** In vitro liver microsome stability and rat hepatocyte/Sprague–Dawley stability assay comparing MC180379 to MC180380. The dog breed was beagle and the mouse microsome strain was CD-1. All sexes (including the hepatocytes) were male except the human microsomes which were mixed gender. Significances were calculated using log-rank (Mantel-Cox) or Student’s t tests. Data are shown as means ± SEMs. *p < 0.05, **p < 0.01, ***p < 0.001

To determine the in vivo antitumor efficacies of MC180379 and MC180380, we tested both enantiomers in three mouse models we previously used to test MC180295, including two colon cancer mouse models and one AML mouse model. We first subcutaneously injected SW48 colon cancer cells into NOD.Cg-Prkdc^scid^ //2rg^tm1Wjl^/SzJ (NSG) mice and treated them with 20 mg/kg IP test drugs every other day (q.o.d) for 10 weeks. Drug treatment significantly delayed tumor growth, compared to vehicle-treated mice. However, there was no statistical difference in tumor size when treating mice with either MC190295 or its two enantiomers (Fig. 3A). Also, we found no survival difference between mice treated with either MC180379 or MC180380 in this model, although the MC180380-treated group did trend toward prolonged survival (Fig. 3B). Both MC180379 and MC1800380 were well-tolerated, with no differences in neutrophil, lymphocyte, or monocyte counts after drug treatment (Additional file 1: Fig. S3A). Interestingly, MC180380 treatment led to slightly higher platelet counts than MC180379 (Additional file 1: Fig. S3B).

We then performed a similar experiment using another colon cancer cell line, HT29. Tumors grew significantly slower after drug treatment (Fig. 3C), with MC180380 extending survival longer than MC180379 (Fig. 3D). We also found that MC180379-treated mice had more severely ulcerated tumors (data not shown), compared to MC180380-treated mice, despite showing no difference in tumor sizes (Fig. 3C). Regarding toxicity, complete blood counts in this model showed no significant neutropenia, lymphopenia, monocytopenia, or thrombocytopenia after drug treatment (Additional file 1: Fig. S3C and S3D). Interestingly, MC180380-treated mice had higher absolute lymphocyte and monocyte counts than MC180379-treated mice. Consistent with the SW48 model, MC180380-treated mice exhibited higher platelet counts than those treated with MC180379 (Additional file 1: Fig. S3C,D). Lastly, we generated an AML mouse model where luciferase-labeled, GFP-positive MV4-11 cells were injected intraperitoneally (i.p.) into NSG mice, followed by drug treatment. Tumor burden, as measured by luciferase intensities, was lower with MC180380 (with better survival), compared to MC180379 treatment (Fig. 3E,F).

To determine why MC180380 was more efficacious than MC180379, we tested both enantiomers' CDK9 inhibitory activities, finding comparable IC50s (11 nM vs. 9 nM) (Additional file 1: Fig. S3E). We then assessed in vitro solubility and metabolism of the two enantiomers. We compared their solubilities in PBS and found no significant difference (53.5 µM v. 52.6 µM) between the two enantiomers (Additional file 1: Fig. S3F). We then tested their in vitro metabolic profiles using liver microsome and rat hepatocyte stability assays. Interestingly, in liver microsomes, we found MC180380 more stable than MC180379 in all four species, with the same result in rat hepatocytes (Fig. 3G). Therefore, MC180380 is more resistant than MC180379 to metabolism, which might explain MC180380's better in vivo efficacy. Additionally, we found that MC180380-treated mice had higher absolute lymphocyte, monocyte, and platelet counts, compared to MC180379-treated mice, indicating that MC180380 is less bone marrow-suppressive (and thus less toxic) than MC180379, in vivo.

### Additivity/synergy with the hypomethylating drug decitabine

**Fig. 4:**
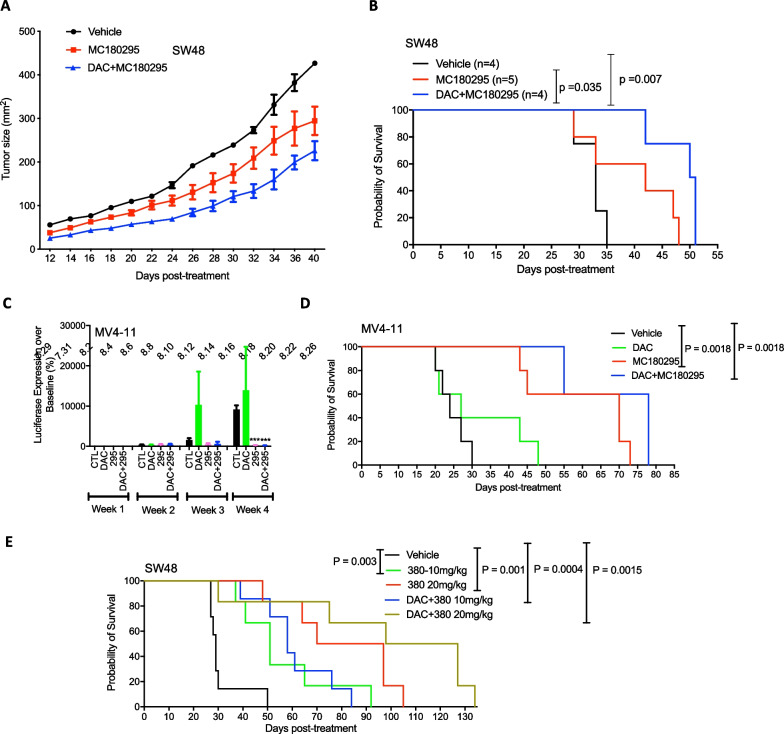
Antitumor and survival-extending properties of a hypomethylating agent (DAC) combined with MC180295. **A** Antitumor effects of MC180295 and MC180295 + DAC. NSG mice were inoculated (s.c.) with 2 × 10^6^ SW48 cells. Eleven days later, when tumors were palpable, 20 mg/kg MC180295 or vehicle were administered (i.p.) q.o.d. 0.5 mg/kg DAC was administered (i.p.) daily. Tumor sizes were measured using a caliper. **B** Mouse survival in days. 20 mg/kg MC180295 was administered (i.p.) q.o.d. 0.5 mg/kg DAC was administered (i.p.) daily. **C** NSG mice were inoculated (i.p.) with 5 × 10^5^ MV4-11-luc cells. Four days later, when substantial tumor burden was evident by bioluminescence imaging, MC180295 (20 mg/kg) or vehicle were administered (i.p.) every other day. 0.5 mg/kg DAC was administered (i.p.) daily. Luciferase expression was quantified and calculated. **D** IP-administered MC180295 and DAC + MC180295 significantly extended survival in MV4-11 model mice. **E** Antitumor effects of MC180380 and MC180380 + DAC. NSG mice were inoculated (s.c.) with 2 × 10^6^ SW48 cells. Eleven days later, when tumors were palpable, 10 mg/kg MC180380, 20 mg/kg MC180380, or vehicle was administered (i.p.) q.o.d. 0.5 mg/kg DAC was administered (i.p.) daily. Mouse survival shown in days. Significances were calculated using log-rank (Mantel-Cox) or Student's t tests. Data are shown as means ± SEMs. ***p < 0.001

In cancer, methylated DNA generally associates with transcriptionally repressive chromatin, leading to gene silencing. Many studies have established that the re-expression of such silenced genes can be achieved by combining different epigenetic therapies, resulting in amplification of gene reactivation [1]. Previously, we found that CDK9 inhibition and decitabine (DAC) treatment elicit similar transcriptional profiles, with many DAC target genes also significantly induced by CDK9 inhibition, supporting their combination [7]. To test this hypothesis, we first used the SW48 mouse colon cancer model, in which MC180295 alone could significantly reduce tumor size and prolong overall survival. We then combined DAC with MC180295, finding that the combination led to more robust tumor regression (Fig. 4A), and a significant survival benefit, compared to MC180295 alone (Fig. 4B). In this model, we did not see significant drug toxicities, noting no significant thrombocytopenia, neutropenia, lymphocytopenia, monocytopenia, or weight loss after drug treatments (Additional file 1: Fig. S4A, B, C). Likewise, using the MV411 AML model, we obtained a similar result, seeing no significant weight loss after drug treatment (Additional file 1: Fig. S4D). In the AML model, DAC alone did not show consistent tumor suppression or a survival benefit (Fig. 4C, D), suggesting that the benefit instead was attributable to at least additive effects between the two drugs. To see if the MC180380 enantiomer showed similar synergy, we combined it with DAC in the SW48 mouse model, finding that 20 mg/kg led to better overall survival than 10 mg/kg. Combining DAC with 20 mg/kg MC180380 was most efficacious in extending survival among all groups (Fig. 4E), and we did not see any significant added drug toxicities, as measured by compete blood counts or decreased body weights (Additional file 1: Fig. S4E, F, G).

### MC180295-mediated anti-tumoral effects are partially CD8 + T cell-dependent

Epigenetic therapy aims to modulate transcriptional programming to affect multiple signaling pathways in immune cells and cancer cells, thus influencing immune cell function and immunotherapy. In addition, epigenetic drugs can induce tumor immunogenic cell death, upregulate various tumor-associated antigens and MHC molecules, and elicit generation of antigen-presenting cells (APCs), leading to enhancement of immune cell priming and effector T cell recognition of tumor cells [9–11]. Epigenetic drugs may also target a variety type of immune cells, reduce generation and accumulation of myeloid-derived suppressor cells (MDSCs) [12], inhibit differentiation and function of Tregs [13–15], and increase production of effector T cell chemokines and activated effector T cells [16].

**Fig. 5:**
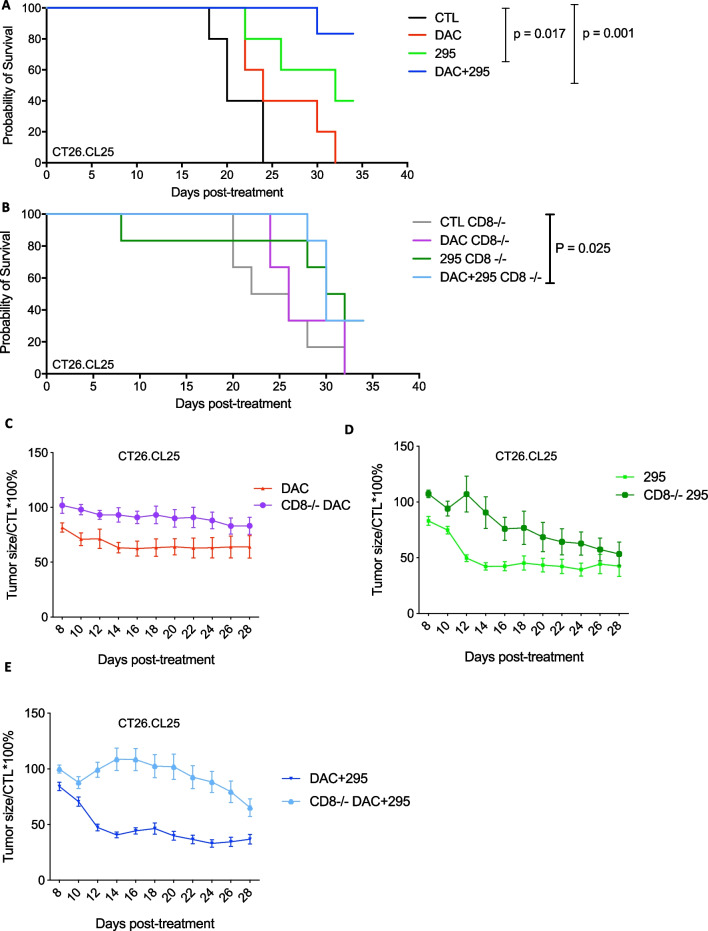
Survival and antitumor effects of MC180295, DAC, or their combination, with/without CD8 + T cells. **A** 20 mg/kg MC180295 was administered (i.p.) q.o.d. 0.5 mg/kg DAC was administered (i.p.) daily. Mouse survival in days. **B** Survival of B6 mice, after CD8 depletion, using an anti-CD8 antibody. 20 mg/kg MC180295 was administered (i.p.) q.o.d. 0.5 mg/kg DAC was administered (i.p.) daily. **C** Antitumor effects of MC180295 with and without CD8 depletion, using an anti-CD8 antibody. B6 mice were inoculated (s.c.) with 5 × 10^5^ CT26.CL25 cells. Eight days later, when tumors were palpable, 20 mg/kg MC180295 was administered (i.p.) q.o.d. 0.5 mg/kg DAC was administered (i.p.) daily. Tumor sizes were measured using a caliper and calculated over vehicle. **D** Antitumor effects of DAC, with and without CD8 depletion, using an anti-CD8 antibody. B6 mice were inoculated (s.c.) with 5 × 10^5^ CT26.CL25 cells. Eight days later, when tumors were palpable, 20 mg/kg MC180295 was administered (i.p.) q.o.d. 0.5 mg/kg DAC was administered (i.p.) daily. Tumor sizes were measured using a caliper and calculated over vehicle. **E** Antitumor effects of DAC + MC180295, with and without CD8 depletion, using an anti-CD8 antibody. B6 mice were inoculated (s.c.) with 5 × 10^5^ CT26.CL25 cells. Eight days later, when tumors were palpable, 20 mg/kg MC180295 was administered (i.p.) q.o.d. 0.5 mg/kg DAC was administered (i.p.) daily. Tumor sizes were measured using a caliper and calculated over vehicle. Significances were calculated using log-rank (Mantel-Cox) or Student's T tests. Data shown as means ± SEMs

Previously, we showed that CDK9 inhibitors upregulate multiple repetitive elements, including endogenous retroviruses (ERVs), and could be combined with immune checkpoint inhibitors, in vivo. We also showed that CDK9 inhibition could increase CD45 + cell numbers and percentages of CD3 + T cells in the tumor environment [7]. To see if CDK9 inhibitory antitumor effects are T-cell dependent, we implanted murine CT26.CL25 colon cancer cells into C57BL/6 mice and tested treatment with DAC and MC180295. These results confirmed that DAC alone could significantly decrease tumor size, in a CD8 + T cell-dependent manner, consistent with a previous report [17] (Fig. 5A–C, Additional file 1: Fig. S5C, and S5D). Likewise, MC180295 significantly decreased tumor size and extended survival (Fig. 5A, D, and Additional file 1: Fig. S5C). However, combining DAC with MC180295 was most efficacious in reducing tumor size and extending survival in CD8 + mice. (Fig. 5A and Additional file 1: Fig. S5C). Mechanistically, depleting CD8 + T cells significantly attenuated the efficacy of epigenetic treatment in this model, lessening tumor reduction and partially inhibiting prolonged overall survival (Fig. 5B and Additional file 1: Fig. S5D). Moreover, DAC antitumor augmentation of MC180295 was largely negated in the CD8- setting (Fig. 5D, E, and Additional file 1: Fig. S5D). It is worth noticing that, over time, the difference between the T cell-depleted groups and controls diminished gradually, suggesting a rapid impact of the drugs on the immune response, and a more gradual epigenetic or cytotoxic effect with continued drug treatment. However, we did not observe significant drug toxicities, with no weight loss, thrombocytopenia, neutropenia, lymphopenia, or monocytopenia, in any treatment groups, in the presence or absence of CD8 + cells (Additional file 1: Figs. S5A, B, E, F, G).

## Discussion

Cyclin-dependent kinases (CDKs) are essential for cell cycling and thus, cancer cell proliferation, making them promising targets for cancer treatment. However, due to similarities in the catalytic sites amongst all CDKs, most CDK inhibitors are non-selective, contributing to narrow therapeutic windows, toxic effects, and unsuccessful clinical trials [6]. Thus, although numerous efforts have been put forth to identify selective CDK inhibitors, only three (the CDK4/6 inhibitors palbociclib, ribociclib, and abemaciclib) have been approved, all for the treatment of advanced hormone receptor-positive breast cancer [18].

Other than CDK4/6, CDKs that regulate transcription are also promising targets. For example, CDK9 inhibition is known to suppress highly expressed genes involved in cancer cell proliferation, metastasis, and treatment resistance, including MYC and MCL-1 [6]. Chromosomal rearrangements in the gene mixed leukemia lineage- 1 (MLL1) are common in leukemias. In particular, MLL1 fusion chimeras enhance recruitment of P-TEFb (transcription elongation factor) to MLL1 target genes, making MLL1-rearranged malignancies very sensitive to CDK9 inhibition [6]. The therapeutic efficacy of CDK9 inhibitors therefore has been reported in MYC-driven [19–22] and MLL1-rearranged [23–25] cancers.

CDK9 inhibition has also been applied in cancers characterized by other oncogenic drivers. For example, CDK9 inhibition can disrupt transcriptional elongation, resulting from BRD4-NUT fusion proteins in NUT midline carcinoma, leading to cancer cell apoptosis [26]. CDK9 inhibition can also decrease phosphorylation of the androgen receptor (AR), decreasing AR-dependent transcription and reducing tumor burden in vivo [27]. In addition, we reported that CDK9 also serves as a transcriptional repressor to maintain gene silencing at heterochromatic loci by phosphorylating the SWI/SNF chromatin-remodeling enzyme BRG1 [7].

Due to the ubiquitous nature of CDKs, the development of CDK9 inhibitors has presented challenges of off-target activity causing increased toxicity without a therapeutic benefit. In that regard, first generation CDK9 inhibitors (flavopiridol, roscovitine, etc.) were pan-CDK inhibitors targeting multiple CDKs. In particular, flavopiridol was a nonselective CDK9 inhibitor (CDK9/cyclin T1 Ki = 3 nM) which showed in vivo activity in hematologic malignancies (e.g., mantle cell lymphoma, CLL) but was halted due to high toxicity [28]. Subsequently, second-generation CDK9 inhibitors (e.g., dinaciclib, AT7519, roniciclib, etc.) were developed, with better selectivity and less side effects. At present, there are approximately 40 CDK inhibitors being developed, in various preclinical and clinical stages [29]. For instance, dinaciclib, a follow-up molecule to flavopiridol, inhibits cell cycle progression in > 100 cancer cell lines [30] and remains in ongoing clinical trials [29].

More recently, a variety of more specific CDK9 inhibitors have been developed and studied in multiple cancers. Among these, AZD-4573 is a highly selective CDK9 inhibitor that induces cancer cell apoptosis and cell death via MCL-1 depletion, thus showing promising preclinical in vivo efficacy, both as a monotherapy and in combination with Venetoclax, in hematologic cancer models [31]. Similarly, CDKI-73 is an orally bioavailable CDK9 inhibitor that can downregulate the anti-apoptotic proteins BCL-2, MCL-1, and XIAP, leading to remarkable antitumor effects in acute myelogenous leukemia (AML) [25], while iCDK9 exhibited more than 600-fold selectivity toward CDK9, compared to CDKs 1, 2, 4, 7, and 8 [32]. VIP-152 is another selective CDK9 inhibitor that shows promising efficacy against hematological malignancies and solid tumors [33, 34]. Using a natural product library in our YB5 cell screen, we recently identified toyocamycin as a novel CDK9 inhibitor and a small molecule tool to modulate CDK9 activity [35].

In this study, we characterized a novel CDK9 inhibitor, MC180295, in multiple cell lines and mouse models. MC180295 showed nanomolar potency and a 20-fold higher selectivity for CDK9, compared to other CDKs. MC180295 was potent against multiple malignancies including melanoma, leukemia, and cancers of the colon, bladder, prostate, and breast. Consistent with other CDK9 studies [29, 36], MC180295 was more active against AML than solid tumors. To better understand MC180295, we separated its enantiomers – MC180379 and MC180380, finding that MC180380 was more potent than MC180379 in terms of gene activation. Interestingly, we found MC180380 to be more stable than MC180379 in liver microsomes and hepatocytes, which might explain its greater drug efficacy. We further found that in different mouse models, MC180379 and MC180380 revealed different antitumor effects. In the SW48 colon cancer model, the two enantiomers showed no difference in tumor size and overall survival. In the HT29 colon cancer model, the two enantiomers showed no difference in tumor size. However, MC180379 led to more severe tumor ulceration and shorter survival than MC180380, and in the MV4-11 AML model, MC180380 was superior to MC180379 in reducing tumor burden and prolonging survival. Overall, the data point to a small but significant advantage for MC180380 over MC180379.

Most cancers are more efficaciously treated with combinations of drugs than monotherapies. Indeed, different combinations of epigenetic drugs have been tested and shown promising results. The most explored combination has been that of DNA methyltransferase (DNMT) and histone deacetylase (HDAC) inhibitors, shown to augment the re-expression of epigenetically silenced genes [1]. This combinatorial paradigm has been explored in many preclinical and clinical studies, with promising results in hematologic and solid tumors [1]. Similar to DNMT/HDAC inhibitors, the CDK9 inhibitor MC180295 could also reactivate genes at nanomolar doses in YB5 cells, with a four-day single dose MC180295 treatment revealing a transcriptional profile similar to DAC treatment. Moreover, most silenced CDK9 target genes were also DAC target genes silenced by DNA methylation [7]. Herein, we showed that in mouse models, DAC could be combined with MC180295 or MC180380 to reduce tumor burden and prolong overall survival, making this combination an attractive anticancer strategy. Analogously, it would be important to explore other epigenetic drugs that could be combined with MC180295 to reactivate silenced genes, and improve survival, in different settings.

Epigenetic drugs can also modulate immune pathways in tumor and immune cells, having been combined with immunotherapy in animal models and clinical studies. For example, DNMT and LSD1 (histone demethylase) inhibitors have been shown to trigger the interferon (IFN) pathway within tumor cells, in part by activation of endogenous retroviruses (ERVs), leading to epigenetic immunosensitization [2, 37]. Here, we showed that responses to CDK9 inhibition were partially dependent on T-cells. Previously, we also showed that CDK9 inhibition, combined with anti-PD1 treatment, synergistically reduced tumor burden, and prolonged survival, in an ovarian cancer model [7]. These data suggest the possible clinical testing of CDK9 inhibition in combination with immune checkpoint inhibitors, or other immunotherapy strategies. Several clinical trials are testing this strategy of epigenetic immunosensitization. In myelodysplastic syndrome, a recent report found significant responses to the combination of a DNMT1 inhibitor and a PDL1 inhibitor in some patients previously resistant to hypomethylating drugs [38]. In bladder cancer previously resistant to immune checkpoint inhibition, the addition of a hypomethylating agent to anti-PD-L1 therapy led to some patients experiencing an immune response and prolonged stable disease, though responses were not seen indicating the need for more efficacious immunosensitization [39]. It would be interesting to clinically test the addition of a CDK9 inhibitor to similar settings.

Lastly, several novel CDK9-binding partners have recently been identified, making them potential targets for combinatorial therapy. For instance, CDK9 was identified as a novel binding partner of the mTOR complex scaffold protein, mLST8, forming distinct complexes in the cytoplasm and nucleus [40]. CDK9 was also found to interact with the chromatin reader KAP1 and the transcription factor SMAD2, in sustaining transcriptional programs involved in cancer maintenance [41]. Additionally, a protein phosphatase 2A complex was shown to antagonize phosphorylation of CDK9 substrates, offering alternative therapeutic opportunities to target transcriptional dysregulation [42].

## Conclusions

Our data show that MC180380 is a CDK9-selective inhibitor with promising antitumor efficacy in mouse models, alone and in combination with DNMT inhibition. Its activity is partially T-cell dependent, further supporting its combination with immunotherapy.

## Methods

### In vitro cellular studies

The live-cell assay for GFP reactivation in YB5 cells was done as previously described [8]. Cells were treated with a single dose of drug 24 h after seeding at low density, and GFP fluorescence measured in a Millipore Guava flow cytometer (EMD, Millipore) instrument, or by imaging in a Cytation instrument (Agilent). Cancer cell lines (listed in Additional file 1: Fig. S1A) were obtained from ATCC or the Temple/Fox Chase Cellular Repository and cultured in the recommended medium. To measure growth inhibition, cells were seeded in 96-well plates at 40% confluency. Fresh medium was changed the next day and drugs were added directly. Drug-free fresh medium was changed on the fourth day. The cells were collected on day 5 by trypsin, suspended in medium, mixed with trypan blue (Thermo Fisher Scientific) (1:1 ratio), and counted using a LUNA II automated cell counter. Each sample was counted at least three times, and average numbers used for the analysis. Each treatment condition was performed in biological triplicates.

### Cyclin-dependent kinase inhibition assays

CDK enzymatic assays were performed by Reaction Biology Corp. (Malvern, PA), using 10 µM ATP for determining MC180295 IC50 values for each CDK family member for which assays were available. Kinase enzymatic assays were also performed by Nanosyn (Santa Clara, CA), using microfluidic technology and MC180295 IC50 curves against 10 CDKs. Selectivity against CDK9 was confirmed for the enantiomers at Reaction Biology Corp. (Malvern, PA).

### Enantiomer separation and characterization

MC180379 and MC180380 enantiomers were separated by Lotus Separations (Princeton, NJ) using Supercritical Fluid Chromatography. Conditions, solvent composition, and column identity are provided below**:** (5 µm, 250 × 20 mm) 15% methanol (0.1% DEA)/CO_2_, 100 bar70 mL/min, 220 nm mL, 8 mg/mL methanol:DCM. *Analytical method:* Chiralcell® OJ-H (5 µm, 250 × 4.6 mm) 20% methanol (DEA)/CO_2_, 120 bar 30 mL/min, 220, 254, and 280 nm inj vol.: 1. The enantiomers were initially obtained as amorphous orange solids. Tentative assignments of stereochemistry were based on the optical rotations of the corresponding exo-2-aminonorbornanes. A single crystal of MC-180380 mesylate, suitable for x- ray diffraction analysis, was produced, and the structure was solved.

The kinetic solubility of test compounds was determined by adapting Millipore® application note protocol AN1730EN00. Liquid handling was performed using PerkinElmer® Verispan and MDT workstations. In triplicate, 4 µL of 10 mM test compounds in DMSO were added to 196 µL of buffer (Dulbecco’s’ PBS or water). The 200 µM compound solutions, in 2% DMSO, were incubated with gentle shaking for 90 min at RT before vacuum filtration in 96-well polypropylene collection plates, using a Millipore® filtration manifold. 160 µL of filtrate was transferred to a 96-well UV Star analysis plate (Greiner Bio-One® plate # 655,801) containing 40 µL acetonitrile. Standard curves were generated by adding 4 µL of 50 × of five concentrations of test compounds, in DMSO, to 40 µL acetonitrile in UV Star plates, followed by 156 µL buffer. Absorbances at 280, 300, 320, 340, and 360 nM were measured using a Molecular Devices® Spectramax Plus microplate reader with Softmax Pro v. 5.4.5 software. Absorbance readings were summed to generate the signal. Test sample concentrations were interpolated from linear standard curves using GraphPad® Prism v 5.04.

### Liver microsome stability assay

Assays were conducted in 96-deep well polypropylene plates. In duplicate, test compounds (1 µM) were incubated in 0.5 mL of 100 mM potassium phosphate buffer (pH 7.4), with 0.5 mg/ml pooled liver microsomes (Life Technologies: CD-1 male mice # MSMCPL; SD male rat # RTMCPL; mixed gender human # HMMCPL. BD Genetest male beagle dog; # 452,601; male marmoset # 452,340), 2 mM tetra sodium NADPH, and 3 mM MgCl_2_, for 60 min in a Labnet® Vortemp incubator at 37 °C, with gentle shaking. At five time points, 75 µL of reaction mixtures were transferred on ice to a 96-shallow well stop plate containing 225 µL acetonitrile with 0.1 µM propafenone. A control reaction (lacking NADPH) was incubated for 60 min at 37 °C to demonstrate the NADPH-dependency of compound loss. Standard curves for test compounds were generated using 5 concentrations (in duplicate) that were processed as above but with no incubation time. Stop plates were centrifuged at 2000xg for 10 min in a Sorvall ST 16 centrifuge, and 170 µL of the supernatants transferred to a Waters® Aquity UPLC (Ultra Performance Liquid Chromatography) 700 µL 96-well sample plate with cap mat. A Waters® Aquity UPLC BEH C18 1.7 µM, 2.1 × 50 mm column, at 40 °C, was used to fractionate 5 µL samples, using a 3 min linear 5 to 95% acetonitrile gradient in 0.1% formic acid with a 0.75 mL/min flow rate. In tandem with UPLC, compound concentrations were analyzed using a Waters® TQ MS mass spectrometer in electrospray-positive mode (source temperature 150 °C; desolvation temperature 450 °C; desolvation flow rate of 900 L/hr). Cone voltages, collision energies, and quantitation were optimized and determined using Waters® QuanOptimize software, with propafenone as an internal standard. MC180379:CV 34; CE 34; mrm 359.15 > 114.91; MC180390 CV 34; CE 28; mrm 359.15 > 114.92. GraphPad® Prism v 5.04 was used for nonlinear fitting of time course data to generate t_1/2_ and CL_int_ (intrinsic clearance) values.

### Rat hepatocyte stability assay

CL_int_ (µL/min/10^6^ cells) estimates were determined using a modified method of McGinnity et al. [43]. Cryopreserved rat (Sprague–Dawley) primary hepatocytes (4 male donors) (Life Tech product RTCS10) were rapidly thawed, diluted with Hank’s balanced salt solution at 37 °C and centrifuged for 5 min at 100 × g. Resuspended cells were stained with 0.016% trypan blue and counted. Cell viability was > 80%. 0.25 mL of a 1 × 10^6^ viable cells/ml cell suspension was added to 16-mm diameter wells of an uncoated polystyrene plate. The prewarmed plate was placed in a 37 °C incubator with 5% CO_2_ atmosphere, with 100 oscillations/min shaking. After 5 min, 0.25 mL of 2 µM test compound, in prewarmed HBSS, was added per well and mixed to start the reactions, with duplicate reactions per compound. Verapamil was used as a positive control, and cell-free reactions served as negative controls. At each time point (0, 5, 15, 30, 60, 90, and 120 min), 50 µL aliquots were transferred to a 96-well stop plate containing 200 µL/well acetonitrile: water (85:15), with 0.1% formic acid and 0.1 µM propafenone. The plate was centrifuged for 10 min at 2300 × g. 150 µL of the supernatant was transferred to a mass spectrometry sample plate. Samples were analyzed by UPLC/MS, as described in the microsome stability protocol. Results are expressed as t_1/2_ (min) and in vitro CL_int_ (µL/min/10^6^ cells).

### In vivo studies in mice

We used two animal models for these analyses: NSG mice xenografted with MV411 myeloid leukemia or HT29 or SW48 colon cancer cell lines, and C57/Black6 (B6) mice xenografted with the CT26.CL25 mouse colon cancer cell line. Experimental protocols were approved by Temple University’s Committee on Use and Care of Animals. The MV4-11-luc cell line was generated by transfecting pFUGW-FerH-ffluc2-eGFP into MV4-11 cells. GFP-positive cells were sorted one week after transfection and expanded for in vivo experiments.

Both female and male NOD.Cg-Prkdcscid Il2rgtm1Wjl/SzJ (NSG) mice were used for the experiments. Age- and gender-matched mice were randomly assigned to each group. 8–10-week-old NSG mice were tail vein-injected with 5 × 10^5^ MV4-11-luc cells. Four days later, at which time substantial tumor burden was evident by bioluminescence imaging, mice were randomized and 20 mg/kg MC180295, MC180379, MC180380, or drug solvent administered (i.p.), every other day, until the endpoint was met. 0.5 mg/kg DAC was administered (i.p.) daily until the endpoint was met. 200 µL of diluted D-luciferin, monosodium salt (Thermo Fisher Scientific) (working concentration: 15 mg/mL) was administered (i.p.) into each mouse, and IVIS imaging performed 5 min after the administration. Natural death or accumulation of ascites fluid were used as endpoints.

For the s.c. SW48 model, 2 × 10^6^ cells were injected into the flanks of randomly assigned, gender-matched 8–10-week-old NSG mice. 20 mg/kg MC180379, MC180380, or MC180295 was injected (i.p.), every other day, when the tumors were palpable, 11 days after engraftment, until endpoints were met. Mice were sacrificed before tumor volumes exceeded 400 mm^2^ (endpoint for this model). For the s.c. HT29 model, 2 × 10^6^ cells were injected into the flanks of randomly assigned, gender-matched, 8–10-week-old NSG mice. 20 mg/kg MC180379 or MC180380 was injected (i.p.), every other day, when the tumors were palpable, 12 days after engraftment, until endpoints were met. Mice were sacrificed before tumors volumes exceeded 400 mm^2^ (used as the endpoint for this model). For the CT26.CL25 model, 5 × 10^5^ cells were injected into the flanks of randomly assigned female 8–10-week-old B6 mice. 20 mg/kg MC180295 was injected (i.p.), every other day, when the tumors were palpable, 8 days after engraftment, until endpoints were met. 0.5 mg/kg DAC was injected (i.p.) daily until an endpoint was met. Mice were sacrificed when they developed serious tumoral ulcerations. CD8 + T cells were depleted using an anti-CD8 antibody (InVivoMAb anti-mouse CD8 (Lyt 2.1), BE0118) on days 4, 5, and 11 after inoculation. MC180295, MC180379, and MC180380 were dissolved in NMP (Fisher Scientific), Captisol® (20% w/v) (CyDex), PEG-400 (Millipore Sigma), and normal saline (PBS) (Corning), at a ratio of 1:4:4:11. NMP was added first, followed by Captisol and PEG-400. PBS was added last. DAC was dissolved in water. In a subset of the experiments, after mice were sacrificed, peripheral blood was collected, and complete blood counts were obtained using Abaxis VetScan HM5.

### Statistical analyses

All in vitro experiments were performed in triplicate, and their results presented as means ± SDs or means ± SEMs. Statistical significance was determined using a two-tailed Student’s t test. Survival curves were plotted using the Kaplan–Meier method, and differences compared using the log-rank test. All data were analyzed using the GraphPad Prism® software. *P* < 0.05 was considered statistically significant.

### Supplementary Information

**Additional file 1: Fig. S1.** MC180295 cancer cell growth inhibition and mouse pharmacokinetics.** A** Quantification of MC180295 IC50 values, against 46 cell lines from six cancer types, from Fig. 1A**. B** Time course of MC180295 plasma levels after 2.5 mg/kg oral or 1 mg/kg IV administration to mice. **C** Time course of MC180295 plasma levels after IP 10 mg/kg MC180295 administration to mice. **D** Time course of MC180295 plasma levels after 1 mg/kg MC180295 IV dosing of rats. **Fig. S2**. Epigenetic activity of MC180295. Re-expression of GFP (measured by a Cytation Imaging Reader), 4 days after single-dose treatment of YB5 reporter cells with MC180379, MC180380, and MC180295. Data are shown as means ± SDs, n = 3. ***p < 0.001. **Fig. S3**. Blood cell effects and solubility of the MC180379 and MC180380 enantiomers. **A** Absolute neutrophil, lymphocyte and monocyte counts were determined by a complete blood cell counter after SW48 model mice were treated with either MC180379 or MC180380. **B** Absolute platelet counts were determined by a complete blood cell counter after SW48 model mice were treated with either MC180379 or MC180380. **C** Absolute neutrophil, lymphocyte, and monocyte counts after HT29 model mice were treated with either vehicle, MC180379, or MC180380. **D** Absolute platelet counts after HT29 model mice were treated with vehicle, MC180379, or MC180380. **E** In vitro activity (IC50, in nM) of MC180295, and its two enantiomers, against CDK9. **F** In vitro drug solubility assay comparing MC180379 with MC180380 in PBS. Data are shown as means ± SEMs (A, B, C) or SDs (D) (Student’s t test). *p < 0.05, **p < 0.01, ***p < 0.001. **Fig. S4**. Toxicity of MC180295, MC180380, or their combination with DAC, in SW48 mice. **A** Absolute platelet counts were determined by a complete blood cell counter after SW48 model mice were treated with vehicle, MC180295, or DAC + MC180295. Data are shown as means ± SEMs. **B** Absolute neutrophil, lymphocyte, and monocyte counts, determined by a complete blood cell counter, after SW48 model mouse i.p. treatment with vehicle, MC180295, or MC180295 + DAC. **C** In the i.p. SW48 mouse model, MC180295 or MC180295 + DAC treatment did not affect NSG mouse body weights. **D** In the i.p. MV4-11 mouse model, DAC, MC180295, or MC180295 + DAC treatment did not affect NSG mouse body weights. **E** In the i.p. SW48 mouse model, MC180380 or MC180380 + DAC treatment did not affect NSG mouse body weights. **F** Absolute platelet counts after SW48 model mice were treated with vehicle, MC180380 or MC180380 + DAC. **G** Absolute neutrophil, lymphocyte, and monocyte counts after SW48 model mice were treated with vehicle, MC180380, or MC180380 + DAC. Data are shown as means ± SEMs (Student’s t test). *p < 0.05. **Fig. S5.** Toxicity and antitumor effects of MC180295, DAC, or their combination, in CT26.CL25 mice. **A** In the i.p. CT26.CL25 immunocompetent mouse model, neither MC180295 nor MC180295 + DAC treatment affected NSG mouse body weights. **B** In the i.p. CT26.CL25 immunocompetent mice, neither MC180295 nor MC180295 + DAC treatment affected NSG mouse body weights, after CD8 depletion. **C** Antitumor effects of MC180295 and MC180295 + DAC. B6 mice were inoculated (s.c.) with 5 × 10^5^ CT26.CL25 cells. Eight days later, when tumors were palpable, 20 mg/kg MC180295 was administered (i.p.) q.o.d. 0.5 mg/kg DAC was administered (i.p.) daily. Tumor sizes were measured using a caliper. **D** Antitumor effects of MC180295 and MC180295 + DAC after CD8 + T cell depletion via an anti-CD8 antibody. B6 mice were inoculated (s.c.) with 5 × 10^5^ CT26.CL25 cells. Eight days later, when tumors were palpable, 20 mg/kg MC180295 was administered (i.p.) q.o.d. 0.5 mg/kg DAC was administered (i.p.) daily. Tumor sizes were measured using a caliper. **E** Absolute platelet counts were determined by a complete blood cell counter after mice were treated with vehicle, MC180295, or MC180295 + DAC in the CT26.CL25 immunocompetent mouse model, in the presence or absence of CD8 + T cells. **F** Absolute neutrophil, lymphocyte, and monocyte counts were determined by a complete blood cell counter after mice were treated with vehicle, MC180295, or DAC + MC180295, in CT26.CL25 immunocompetent mice. **G** Absolute neutrophil, lymphocyte, and monocyte counts after mice were treated with vehicle, MC180295, or DAC + MC180295 in CT26.CL25 immunocompetent mice, after CD8 depletion. Data are shown as means ± SEMs

## Data Availability

Available upon request.
