# Supplementary material for: MC180295 is a highly potent and selective CDK9 inhibitor with preclinical in vitro and in vivo efficacy in cancer
Source: Clin Epigenetics. 2024 Jan 3;16:3. doi: 10.1186/s13148-023-01617-3 (PMC10765884; doi:10.1186/s13148-023-01617-3)

Supplemental Figure 1

A

		IC50 (nM)
Colon	CT26.CL25	166
Colon	RKO	76.77
Colon	HT29	75.6
Colon	KM12	102.2
Colon	LOVO	199
Colon	SW48	98.1
Colon	HCT116	92.2
Colon	SW480	153.8
Colon	CaCO2	106.8
Bladder	UC3	101
Bladder	UC6	143
Bladder	UC16	77
Leukemia	Jurkat	141
Leukemia	MV4-11	28
Leukemia	MOLM-13	44
Leukemia	THP-1	43
Leukemia	OCI-AML3	95.3
Leukemia	J-Tag	112
Leukemia	HL-60	120.6
Leukemia	KG-1a	82.3
Leukemia	Raji	136.8
Leukemia	K562	501.8
Leukemia	KG-1	148.4

		IC50 (nM)
Prostate	DU145	211
Prostate	PC3	213
Prostate	LnCap	109
Breast	HCC2218	70.6
Breast	BT549	290
Breast	MCF7	177
Breast	Cama-1	624
Breast	BT474	80
Breast	HTB126	92
Breast	SKBR3	122
Breast	HCCC1937	124
Melanoma	A375	189
Melanoma	VACCC257	343.4
Melanoma	M14	304.9
Melanoma	HS936T	390.8
Melanoma	A2058	197.8
Melanoma	sk-mel28	421.6
Melanoma	451LU	109.6
Melanoma	WM983B	249.9
Melanoma	WM88	102.8
Melanoma	MALME-3M	350.5
Melanoma	WM3918	149.9
Melanoma	WM3912	118.9

B

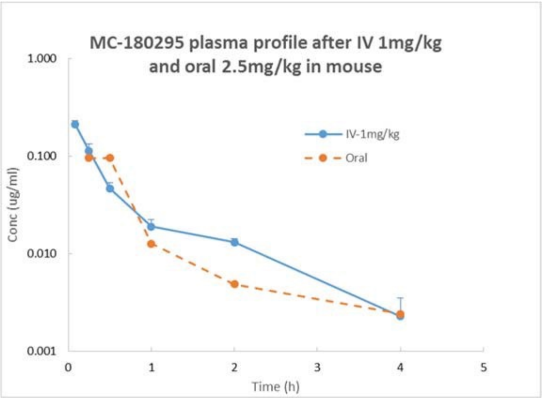

C

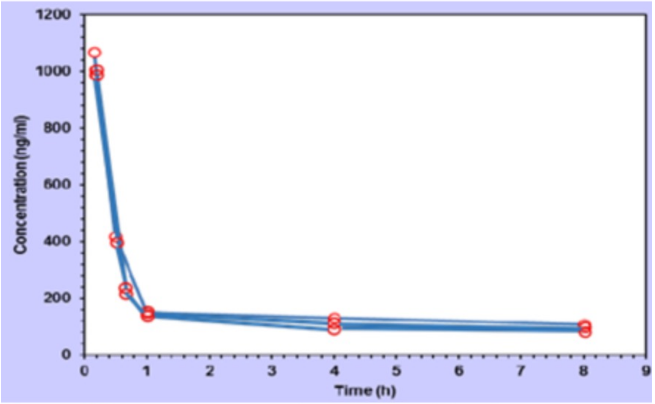

D

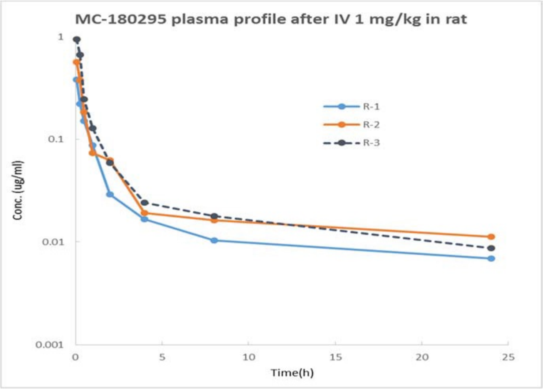

A

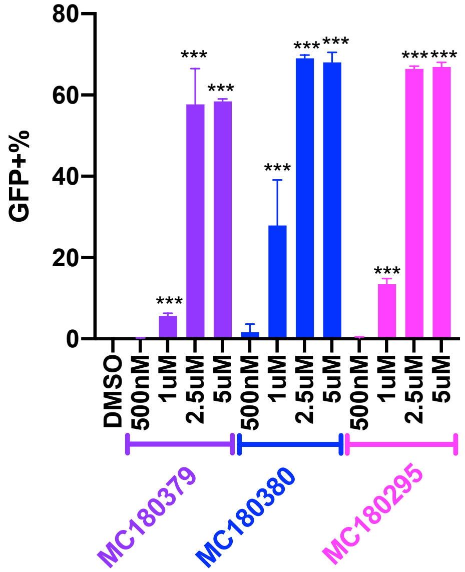
80
60
40
20
0
GFP+%
***
***
***
***
***
***
***
***
***
***
DMSO
500nM
1uM
2.5uM
5uM
500nM
1uM
2.5uM
5uM
500nM
1uM
2.5uM
5uM
MC180379
MC180380
MC180295

Supplemental Figure 3

A

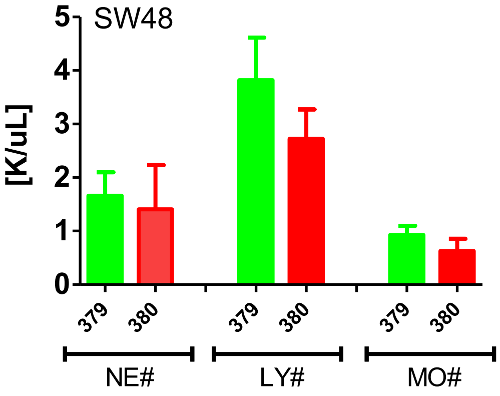

B

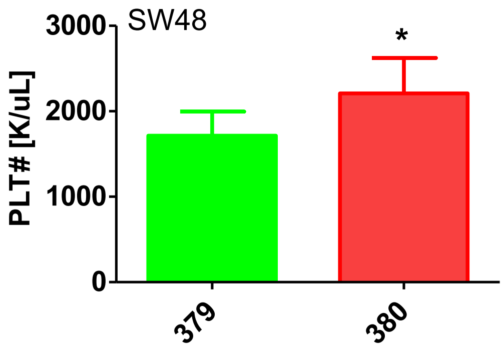

C

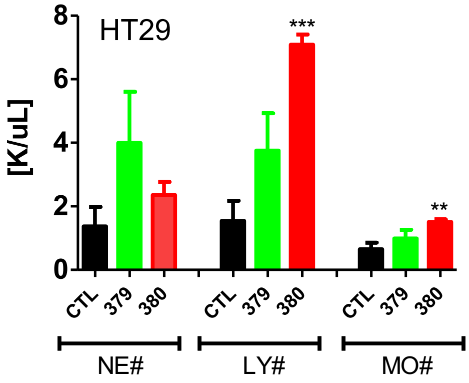

D

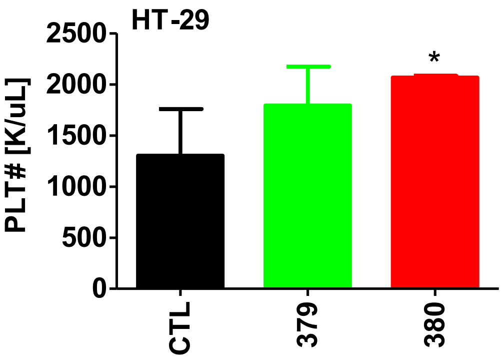

E

	CDK9 IC50 (nM)
MC180295	11
MC180379	11
MC180380	9

F

Compound	Salt	Solvent	Solubility (µM)
MC180379	Free Base	PBS (pH 7.4)	53.5
MC180380	Free Base	PBS (pH 7.4)	52.6

Supplemental Figure 4

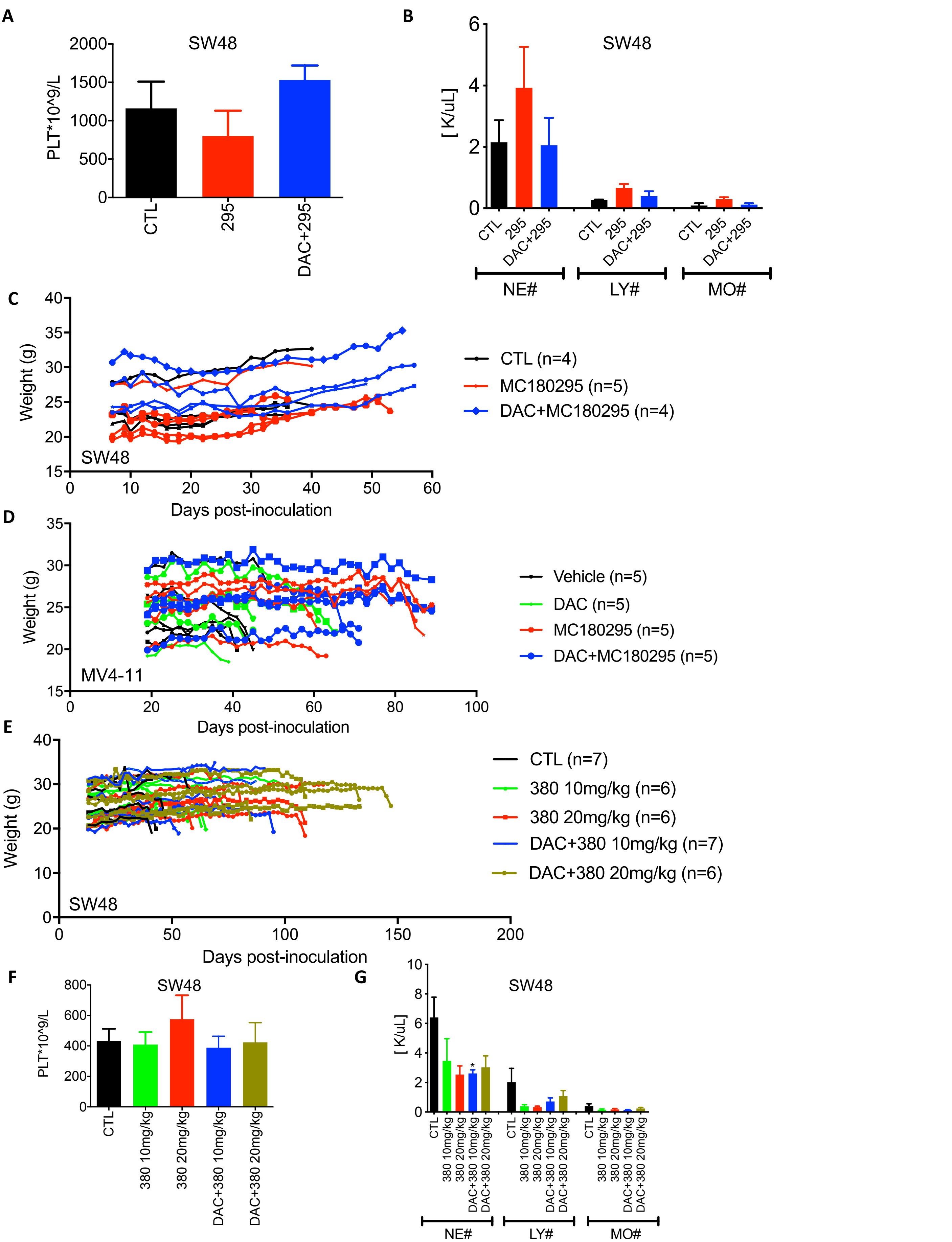

Supplemental Figure 5

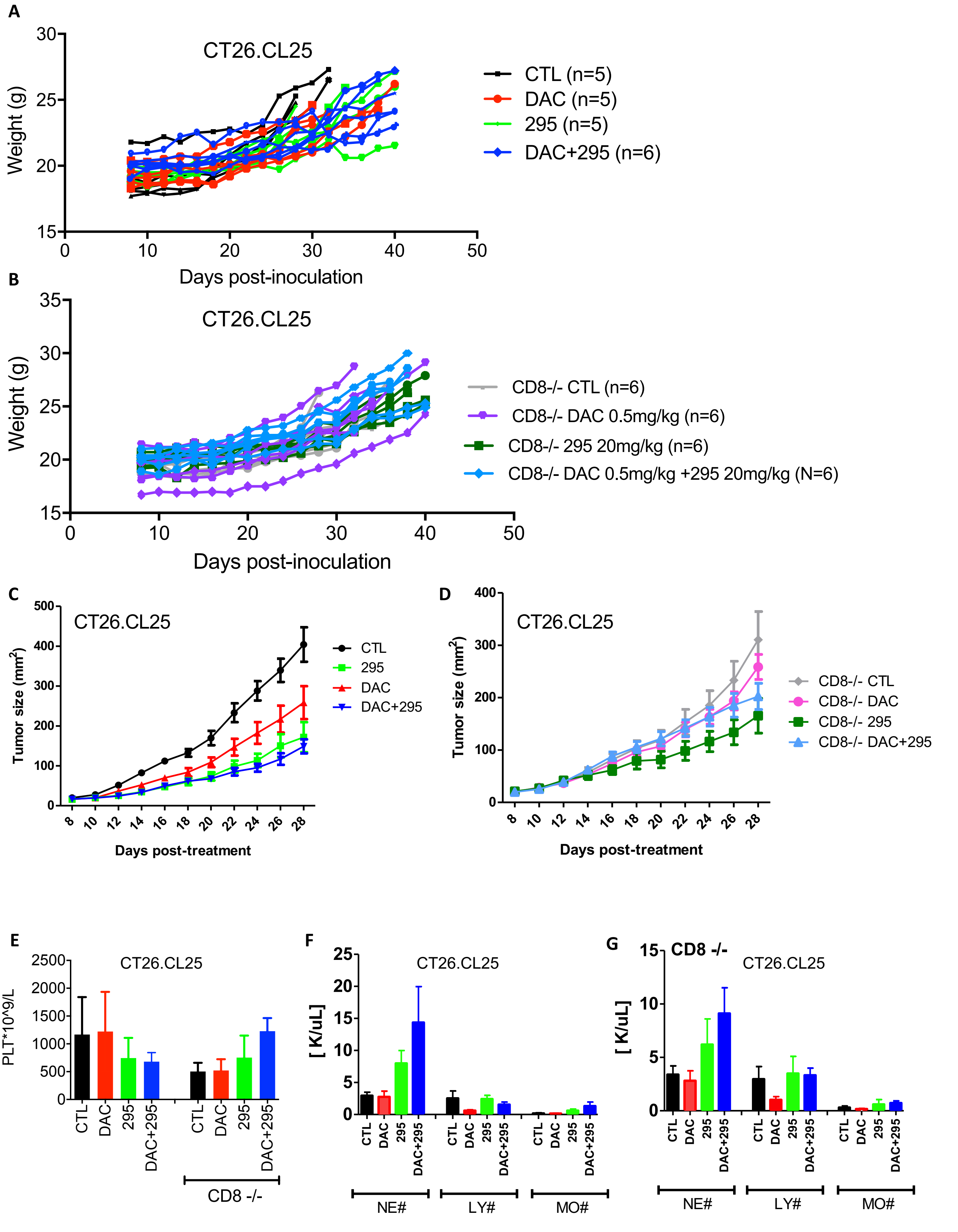

Supplement: Supplementary file 1 — Additional file 1: Fig. S1. MC180295 cancer cell growth inhibition and mouse pharmacokinetics. A Quantification of MC180295 IC50 values, against 46 cell lines from six cancer types, from Fig. 1A. B Time course of MC180295 plasma levels after 2.5 mg/kg oral or 1 mg/kg IV administration to mice. C Time course of MC180295 plasma levels after IP 10 mg/kg MC180295 administration to mice. D Time course of MC180295 plasma levels after 1 mg/kg MC180295 IV dosing of rats. Fig. S2. Epigenetic activity of MC180295. Re-expression of GFP (measured by a Cytation Imaging Reader), 4 days after single-dose treatment of YB5 reporter cells with MC180379, MC180380, and MC180295. Data are shown as means ± SDs, n = 3. ***p < 0.001. Fig. S3. Blood cell effects and solubility of the MC180379 and MC180380 enantiomers. A Absolute neutrophil, lymphocyte and monocyte counts were determined by a complete blood cell counter after SW48 model mice were treated with either MC180379 or MC180380. B Absolute platelet counts were determined by a complete blood cell counter after SW48 model mice were treated with either MC180379 or MC180380. C Absolute neutrophil, lymphocyte, and monocyte counts after HT29 model mice were treated with either vehicle, MC180379, or MC180380. D Absolute platelet counts after HT29 model mice were treated with vehicle, MC180379, or MC180380. E In vitro activity (IC50, in nM) of MC180295, and its two enantiomers, against CDK9. F In vitro drug solubility assay comparing MC180379 with MC180380 in PBS. Data are shown as means ± SEMs (A, B, C) or SDs (D) (Student’s t test). *p < 0.05, **p < 0.01, ***p < 0.001. Fig. S4. Toxicity of MC180295, MC180380, or their combination with DAC, in SW48 mice. A Absolute platelet counts were determined by a complete blood cell counter after SW48 model mice were treated with vehicle, MC180295, or DAC + MC180295. Data are shown as means ± SEMs. B Absolute neutrophil, lymphocyte, and monocyte counts, determined by a complete blood cell counter, aft [file 13148_2023_1617_MOESM1_ESM.pdf]
